# Culture of Mouse Embryonic Stem Cells with Serum but without Exogenous Growth Factors Is Sufficient to Generate Functional Hepatocyte-Like Cells

**DOI:** 10.1371/journal.pone.0023096

**Published:** 2011-08-02

**Authors:** Karen Pauwelyn, Philip Roelandt, Tineke Notelaers, Pau Sancho-Bru, Johan Fevery, Catherine M. Verfaillie

**Affiliations:** 1 Stem Cell Institute Leuven, Catholic University Leuven, Belgium; 2 Department of Hepatology, University Hospitals Leuven, Leuven, Belgium; 3 Liver Unit, Hospital Clinic, Institut d'Investigacions Biomedicale August Pi i Sunyer (IDIBAPS), CIBERehd, Barcelona, Spain; University of Southern California, United States of America

## Abstract

Mouse embryonic stem cells (mESC) have been used to study lineage specification *in vitro*, including towards a hepatocyte-like fate, and such investigations guided lineage differentiation protocols for human (h)ESC. We recently described a four-step protocol to induce hepatocyte-like cells from hESC which also induced hepatocyte-like cell differentiation of mouse induced pluripotent stem cells. As ESC also spontaneously generate hepatocyte-like cells, we here tested whether the growth factors and serum used in this protocol are required to commit mESC and hESC to hepatocyte-like cells. Culture of mESC from two different mouse strains in the absence of serum and growth factors did not induce primitive streak/definitive endoderm genes but induced default differentiation to neuroectoderm on day 6. Although Activin-A and Wnt3 induced primitive streak/definitive endoderm transcripts most robustly in mESC, simple addition of serum also induced these transcripts. Expression of hepatoblast genes occurred earlier when growth factors were used for mESC differentiation. However, further maturation towards functional hepatocyte-like cells was similar in mESC progeny from cultures with serum, irrespective of the addition of growth factors, and irrespective of the mouse strain. This is in contrast to hESC, where growth factors are required for specification towards functional hepatocyte-like cells. Culture of mESC with serum but without growth factors did not induce preferential differentiation towards primitive endoderm or neuroectoderm. Thus, although induction of primitive streak/definitive endoderm specific genes and proteins is more robust when mESC are exposed to a combination of serum and exogenous growth factors, ultimate generation of hepatocyte-like cells from mESC occurs equally well in the presence or absence of exogenous growth factors. The latter is in contrast to what we observed for hESC. These results suggest that differences exist between lineage specific differentiation potential of mESC and hESC, requiring optimization of different protocols for ESC from either species.

## Introduction

Development of the mammalian liver is the result of a multistep process, requiring different growth factors at the correct concentrations, location and time, aside from specific cell-cell and cell-extracellular matrix interactions [1]. Understanding these molecular signals favouring liver development is thought to be essential to develop *in vitro* differentiation protocols to induce a hepatic fate in ESC. Although ESC from human and murine origin are pluripotent, it is becoming clear that species differences, and likely also genetic differences within a given species, influence the culture requirements as well as the efficiency of lineage differentiation of different ESC lines.

**Figure 1:**
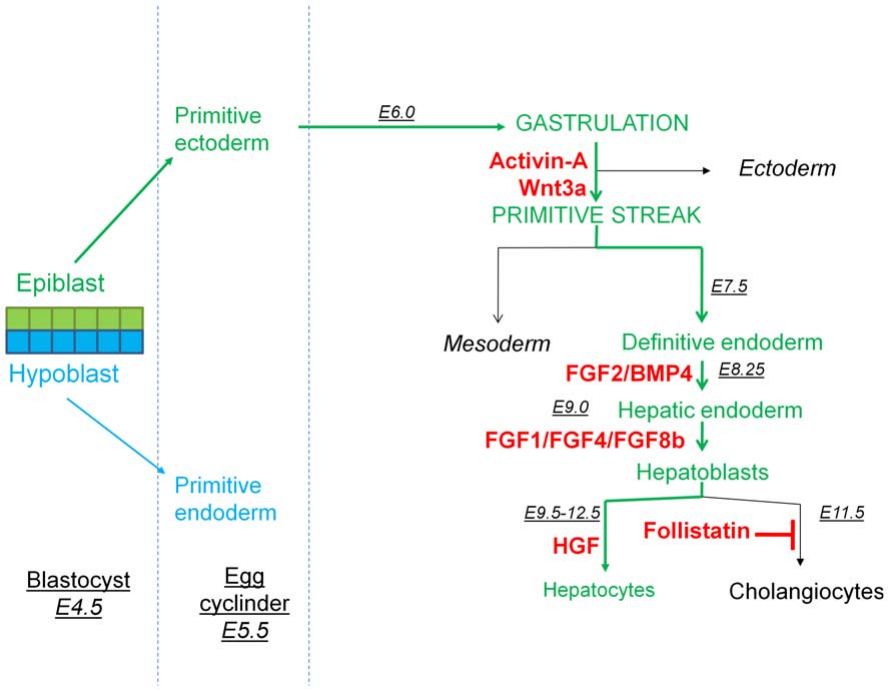
Overview of liver embryogenesis. Growth factors used to mimic different developmental step are indicated in red.

Liver development can be divided in 4 consecutive steps: gastrulation of the epiblast with the formation of first primitive streak (PS) and then definitive endoderm (DE); specification of DE into foregut, midgut and hindgut endoderm [2], [3]; subsequent specification of the ventral foregut endoderm towards a hepatic cell fate [4]; and finally, outgrowth of the liver bud and terminal differentiation of hepatoblasts into either hepatocytes or cholangiocytes [1], [5] (Figure 1). Based on this knowledge, we recently described a 4-step differentiation protocol that supports the differentiation of hESC lines [6] towards DE and functional hepatocyte-like cells.

To differentiate mESC to hepatocytes, some groups have used in the initial step of differentiation embryoid body cultures, wherein mESC spontaneously start to express a wide range of genes of different lineages, including definitive endoderm, but also primitive endoderm (PrE) [7]. Multiple studies have used sequential addition of growth factors in 2D cultures, as in the protocol described by us for hESC [6], to differentiate ESC towards hepatocyte-like cells [8], [9], [10]. However, most studies did not assess whether the growth factors added enhance the induction towards liver fate above the normal spontaneous ability of mESC to generate endoderm and cells with hepatocyte features. In addition, few if any study directly compared the ability of a given protocol to induce hepatic differentiation from mESC as well as hESC, or mESC from different genetic background.

Here, we tested if the different cytokine cocktails used sequentially to induce hESC to cells with hepatocyte-like features are required to induce hepatocyte-like cells from mESC originating from C57Bl/6 and 129 mice.

## Results

### Serum or Activin-A/Wnt3a is sufficient to induce primitive streak and definitive endoderm from mESC

**Figure 2:**
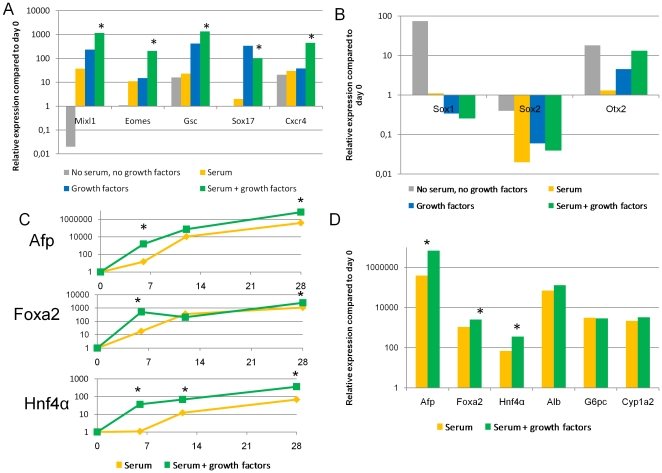
Gene expression in mESC-R1 during different stages of mesendoderm and hepatic specification during the 20 day differentiation process (n = 3). Relative expression values on day 6 compared to day 0 of mesendodermal [A] and neurectodermal genes [B] in the different conditions. Time-dependent gene expression of hepatic endoderm specific genes [C] and relative expression values on day 20 compared to day 0 of hepatocyte-specific genes [D]. *p<0.05 serum+growth factors versus serum only.

**Figure 3:**
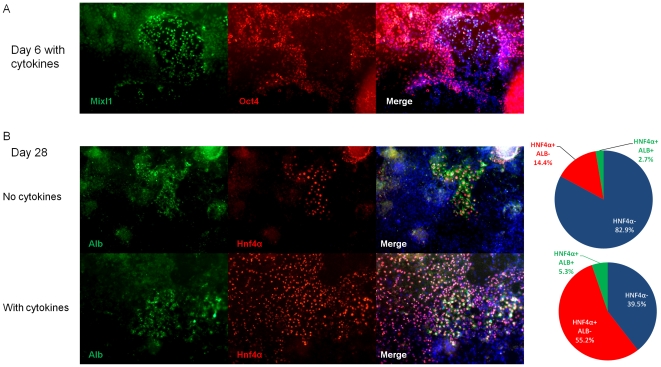
Immunofluorescence assessment mESC progeny (n = 3). [A] Presence of Oct4−/Mixl1+ cells on day 6, only in the presence of additional growth factors. In the condition without growth factors, no Oct4−/Mixl1+ cells could be observed (data not shown). [B] Presence of Hnf4α+/Alb+ cells on day 20, was significantly higher in the condition with additional growth factors, as quantified in the pie-charts.

In the absence of serum or growth factors minimal induction of PS/DE genes was observed by day 6. Moreover, the absence of both serum and growth factors resulted in massive cell death by day 10. However, the addition of serum alone (3 different batches), Activin-A and Wnt3a alone, or a combination of all induced a significant increase in the expression of the PS/DE genes (*Mixl1*, *Gsc*, *Cxcr4*, *Brachyury* and *Eomes*) by day 6 in both mESC lines from 129 and C57Bl/6 mice. The highest level of gene induction was seen when cells were cultured with the combination of serum plus Activin-A/Wnt3a, followed by Activin-A/Wnt3a alone, and then serum alone (Figure 2A). Noteworthy, expression levels of PS/DE genes decreased following day 6 in both cell lines, whether the cells were treated with Activin-A and Wnt3a or not (Table S1). No significant differences were seen in expression levels of the pluripotency gene *Oct4* between the different treated groups. These results are consistent with the notion that TGF-β family members and Wnt's are present in serum [11] at levels sufficient to induce PS/DE genes. In addition, serum may contain additional factors that enhance the effect of Activin-A/Wnt3a on PS/DE induction. Consistent with the highest expression of PS/DE genes by RT-qPCR, Mixl1^+^Oct4^−^ cells could only be identified by immunohistochemistry on day 6 in mESC progeny treated with both serum and Activin-A/Wnt3a (Figure 3A).

**Figure 4:**
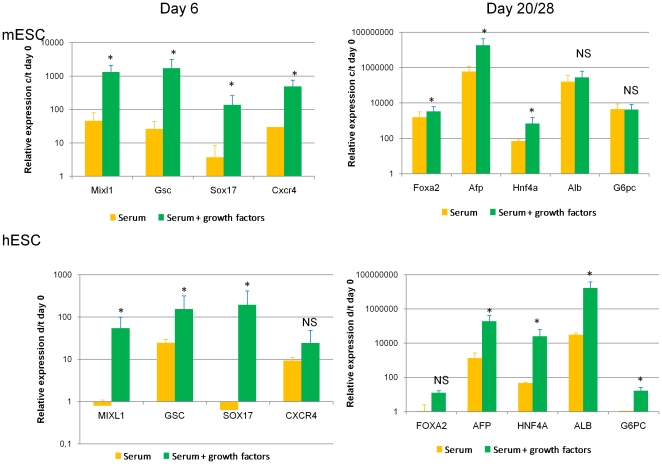
Comparison of gene expression between hESC-H9 and mESC-R1 progeny (n = 3, *p<0.05, NS = not significant). Gene expression of mesendodermal and hepatocyte-specific genes during hESC and mESC differentiation.

In contrast to the findings in mESC, when hESC were cultured with serum but without Activin-A/Wnt3a minimal to no induction of PS/DE specific transcripts were seen on day 6, while these transcripts were induced significantly in the presence of the two cytokines (Figure 4).

### Serum or Activin-A/Wnt3a is sufficient to prevent default differentiation of mESC to neuroectoderm and primitive endoderm

Default differentiation of mESC to neuroectoderm occurs when culturing mESC in serum-free medium and in the absence of any growth factors [12], [13]. We demonstrated that *Sox1* was significantly up-regulated when differentiations were initiated in the absence of serum while presence of serum or growth factors prevented induction of *Sox1* expression in mESC (Figure 2B). Of note, levels of *Otx2* were significantly increased in both mESC cell lines cultured with serum and Activin-A/Wnt3a (n = 3, p<0.05), most likely reflecting the important role of *Otx2* during gastrulation, rather than neural differentiation [14], [15].

Previous studies have also shown a preferential differentiation of mESC towards primitive and visceral endoderm (PrE/VE) (*Thbd*, *Sox7*, *Tmprss2*, *Hnf4α*, *Ttr*, *Afp*) when mESC are cultured in 2D without Activin-A [16], [17]. However, on day 6, expression levels for *Thbd* and *Tmprss2* were identical in mESC-progeny cultured with or without Activin-A/Wnt3a in the presence of serum. *Sox7* levels were identical or even higher in the Activin-A/Wnt3a treated mESC compared with serum alone controls (Table S1).

### Addition of sequential cytokine cocktails does not affect final expression levels of mature hepatic genes in mESC progeny

In both mESC cell lines, *Sox17*, *Afp*, *Ttr*, *Foxa2* and *Hnf4α* (genes expressed in hepatic endoderm) increased significantly already from day 6 onwards when differentiation was induced with a combination of growth factors and serum (n>3), whereas induction of these genes did not occur until day 12 in the no-cytokine condition (n = 3) (Figure 2C). However, by day 28, expression levels of these genes were similar in mESC progeny irrespective of the addition of growth factors, with the exception of *Hnf4α* (n = 3). During further differentiation, a progressive increase in expression of transcripts for *Alb*, *Aat*, *Tat*, *G6p*, *Pepck*, *Cyp7a1* and *Cyp1a2* was observed in both cell lines irrespective of whether growth factors were added or not (n = 3) (Figure 2D, Table S1).

Again in contrast to mESC, the final progeny of hESC-derived hepatocyte-like cells' expression of hepatoblast and more mature hepatocyte-genes was significantly higher in cells differentiated in the presence of growth factors, compared with cells treated with serum alone (Figure 4).

### Growth factors do not affect expression of liver-specific non-parenchymal genes and other mesodermal genes in mESC progeny

Because the liver not only consists of hepatocytes, we tested whether mESC also generate cells with features of hepatic sinusoidal endothelial cells (*Lyve1*, *Stab2*, *Vap1*, *CD32b*, *Mrc1*, *Tie2*, *Ve-cadherin*) and hepatic stellate cells (*Alcam*, *Col1a1*, *Desmin*, *Gfap*, *αSma*, *Gpr91*, *Crbp1*) [18], [19]. A significant increase in *Tie2*, *Ve-cadherin* and *Lyve1* transcripts was seen during differentiation (∼2^5–10^ times increase), irrespective of cytokine addition. Upon differentiation of both cell lines, expression of *Alcam*, *Col1a1*, *Desmin*, *Gfap*, *αSma* and *Crbp1* was significantly up-regulated by day 28 as compared to day 0 (p<0.05), but again irrespective of cytokine addition. We also assessed the expression of other mesodermal genes (*Cnn1*, *Sm22*, *Nkx2-5*, *Mesp2*, *Osterix*), but these were not induced whether or not growth factors were added (Table S1).

### Minimal differences in expression of cytokine transcripts between mESC cultured with or without growth factors

Because we observed only minimal differences between differentiations done with or without addition of exogenous growth factors, we determined if the growth factors added sequentially might be expressed endogenously by the progeny of mESC themselves. To induce PS/DE, a combination of Activin-A and Wnt3a is used. Fgf8 is a known downstream target of Wnt signalling and its induction is required for PS formation and migration of cells into the PS [20], whereas Foxh1 combined with Smad2/3/4 are important for the amplification of Nodal expression before and during gastrulation [21]. *Fgf8* was more induced at day 6 in the presence of Activin-A and Wnt3a compared with serum alone, consistent with the observation that the PS/DE genes were most robustly expressed in this condition. *Nodal*, already highly expressed in undifferentiated mESC, persisted longer in the presence of Activin-A and Wnt3a in mESC, but levels of *Foxh1* were identical in both conditions. In undifferentiated hESC, the levels of *NODAL* were lower compared to undifferentiated mESC (relative expression 0.2% of GAPDH in hESC versus 5.8% of Gapdh in mESC), but were induced to levels similar to those found in mESC on day 6 when Activin-A/Wnt3a were used to induce differentiation from hESC. We also assessed the expression of the different *Fgf'*s, *Bmp4* and *Hgf*, growth factors added in steps 2–4, which were expressed to a similar degree in both conditions tested and in both mESC lines (Table S1).

### Growth factors increase the number of Hnf4α^+^ cells in mESC progeny significantly

As no major differences were observed between the 2 mESC lines, we performed immunochemistry and functional assays only on the R1 cell line (n≥3). By day 28, Alb^−^Hnf4α^+^ (endoderm, mostly likely hepatic endoderm) and Alb^+^Hnf4α^+^ cells (hepatoblasts/hepatocytes) could be detected whether or not growth factors were used during differentiation (Figure 3B). Consistent with the RT-qPCR results, a significantly higher number of Hnf4α^+^ cells could be observed when differentiations were done with growth factors (60.5±12.0% versus 17.1±14.7% no growth factors, p<0.0001). Similarly a small but significant increased number of Alb^+^ cells was detected when differentiations were performed under guidance of growth factors (5.3±2.4% of total cells versus 2.7±3.2% no growth factors, p<0.01). Compared to serum-only condition, the addition of growth factors increased the number of Hnf4α^+^ cells by 3.5-fold, while the number of Alb^+^ cells was only increased by 2-fold. This suggests that the addition of growth factors during mESC differentiation enhances (hepatic) endoderm formation to a greater extent than hepatocyte maturation.

### Final mESC progeny has similar hepatic function irrespective of cytokine addition

**Figure 5:**
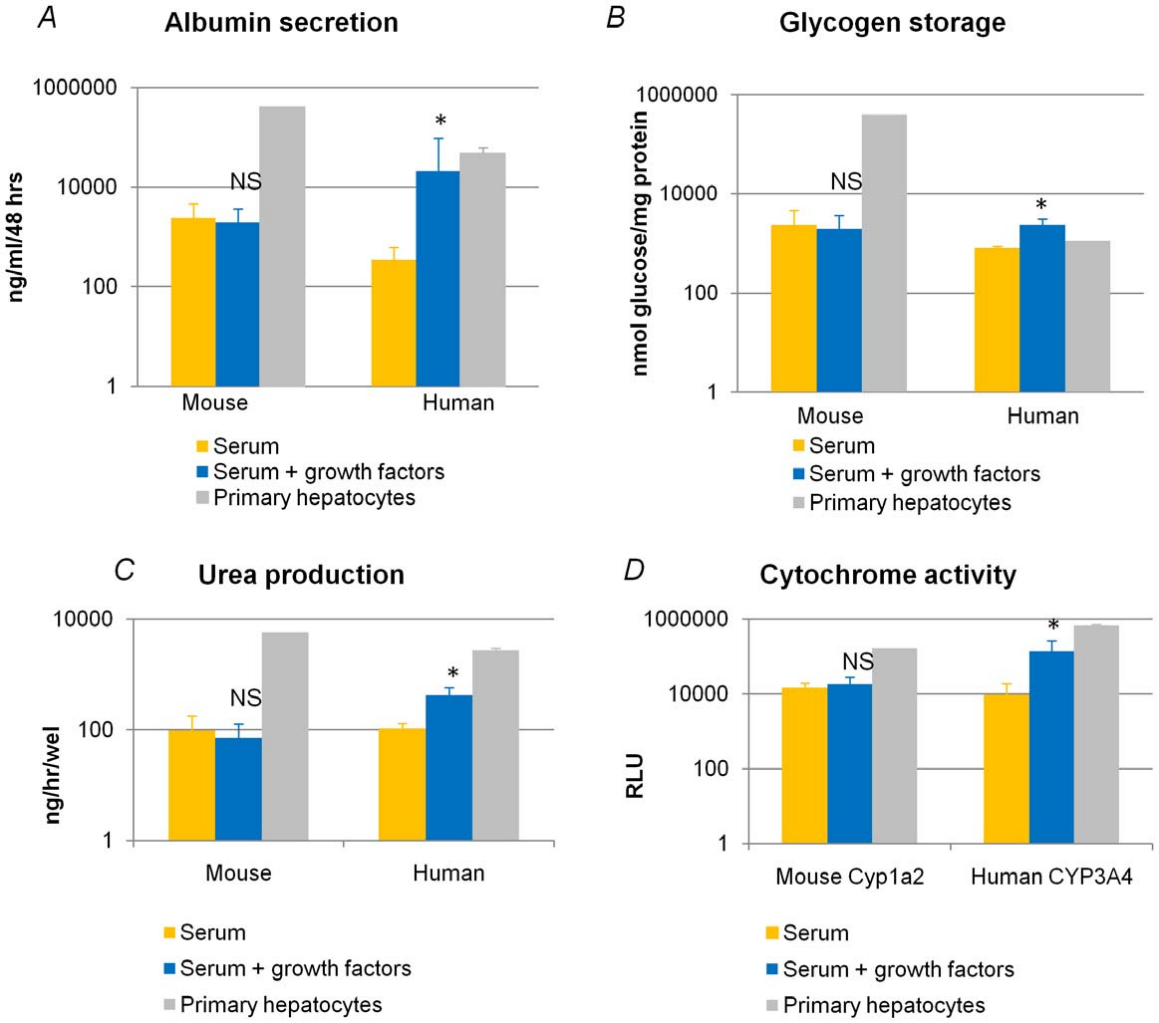
Functional characteristics of mESC and hESC progeny as well as primary hepatocytes at day 20 (n = 3, *p<0.05, NS = not significant). [A] Albumin secretion (ng/ml/48 hrs) [B] Storage of glycogen (nmol glucose/mg protein) [C] Urea production [D] Cytochrome P450 activity. In mESC progeny no significant differences were observed between the two conditions, while in the hESC progeny functional capacities were significantly higher in the presence of additional growth factors.

Consistent with the similar levels of *Alb* transcripts during differentiation and the presence of 5.2 and 2.4% Alb^+^ cells in progeny of mESC cultured with or without growth factors, respectively, similar amounts of albumin were secreted in the culture supernatants at day 28 of mESC progeny differentiated with or without growth factors (Figure 5A). Likewise, accumulation of glycogen, urea production (and increase after NH_4_HCO_3_) and Cyp1a2 function was detected and similar in both conditions (Figure 5B–D). The functional capacity of either progeny was between 0.24% and 5.8% of mature mouse hepatocytes.

Consistent with the lower mature hepatocyte transcript expression found in hESC differentiated without addition of growth factors compared with hESC differentiated with growth factors, differences could also be noticed at the functional level. Albumin secretion, accumulation of glycogen, urea production (and increase after exposure to NH_4_HCO_3_) and CYP3A4 function were significantly higher in hESC progeny generated in cytokine supplemented differentiations than progeny of hESC allowed to differentiate without addition of growth factors (Figure 5A–D).

## Discussion

Understanding the mechanisms that regulate hepatic epithelial cell differentiation are thought to be essential for the creation of efficient, programmed hepatic differentiation protocols from pluripotent stem cells. We here demonstrate that differences in response of pluripotent stem cells to cytokine-mediated lineage specification and differentiation between species will need to be taken into account, when inducing hepatic differentiation from ESC.

During development of the mammalian liver, posterior epiblast cells from the blastocyst at first undergo a process called gastrulation, which results in the formation of mesendoderm (ME), followed by DE and mesoderm. DE gives rise to the foregut, midgut and hindgut endoderm. The liver develops from the foregut endoderm, in response to factors secreted by the adjacent cardiac mesoderm (Fgf1/2) and the septum transversum mesenchyme (Bmp2/4). The foregut endoderm forms the liver bud, which contains bipotential hepatoblasts. Proliferation of the newly specified early hepatoblasts can be increased by other FGF's [5].

Induction of DE from mESC was initially described by embryoid body formation either alone or combined with growth factors mimicking development [22], [23], [24]. More recently many groups have used monolayer cultures to induce DE and subsequently hepatic endoderm in mESC [16], [17], [25]. These studies have demonstrated the importance of initial cell density, presence of serum, and the presence of Activin-A (>10–50 ng/ml), for PS/DE induction [16], [17], [23]. In addition, DE commitment from mESC and hESC in monolayer cultures appears to be enhanced by an inhibitor of GSK-3β or by *Wnt3a* conditioned medium [26].

We described a protocol that supports directed differentiation of hESC in monolayer culture supplemented with 2% FCS to hepatocyte-like cells by sequential induction of PS/DE by Activin-A and Wnt3a, definitive and hepatic endoderm by BMP4 and FGF's, and hepatocyte-like cells with HGF and Follistatin (Figure 1) [6]. Using the same hepatic differentiation protocol, but extending step 4 to 28 days, we here demonstrate that mESC cells from 129 and C57Bl/6 mice can be differentiated towards functional hepatocyte-like cells. Genes, characteristic for PS/DE were maximally and transiently expressed in response to Activin-A and Wnt3a on day 6, followed by formation of hepatic endoderm and finally gradual hepatic maturation. Hepatic maturation was similar in both mESC lines. The results of the functional assays (0.24 to 5.8% of primary hepatocytes activity) were comparable with the functional results obtained when applying the protocol to human ESC [6].

However, although the exogenous addition of growth factors greatly improves PS/DE formation, as well as final hepatic commitment from hESC, this was not true for mESC. mESC cultured with serum but without supplementation of growth factors, differentiate to hepatocyte-like cells, with functional properties comparable to those obtained when differentiation was guided by growth factors. However, induction of transcripts of PS/DE stage cells was significantly higher in the cultures containing Activin-A and Wnt3a as was the expression of hepatic endoderm genes, suggesting that exogenous addition of growth factors enhances and accelerates the formation of hepatic endoderm. This notion is strengthened by the higher number of Mixl1^+^/Oct4^−^ cells found on d6, and the percent Hnf4α^+^ cells on d28 in cytokine treated mESC progeny.

The finding that simple addition of serum leads to PS/DE gene expression is consistent with the notion that TGF-β family members and Wnt's are present in serum, apparently at levels sufficient to induce PS/DE genes in mESC. One difference between mESC and hESC is the expression levels of *Nodal/NODAL* and *Foxh1/FOXH1*, which are significantly higher expressed in mESC than hESC. As Nodal is the *in vivo* counterpart of Activin, endogenously produced Nodal may support the PS/DE induction in mESC progeny cultured without exogenous Activin-A. It is worth noting that the combination of growth factors and serum induced PS/DE genes most robustly, suggesting that additional factors in serum, other than TGF-β members and Wnt's, may play a role in for PS/DE induction. ‘Spontaneous’ maturation of hepatic endoderm and hepatoblasts once the cells go through the PS/DE, may be the result of endogenous production of growth factors as assessed by RT-qPCR (*Bmp4*, *Fgf2*, *Fgf4*, *Fgf8*, till d12, and low levels of *Hgf* from d12 onwards).

In contrast to previous studies [16], [17], [27], no preferential differentiation of mESC to PrE was seen in the absence of Activin-A and Wnt3a. Although some genes, typically expressed in PrE, such as *Sox7*, *Thbd* and *Tmprss2*, became expressed to significant higher levels compared to undifferentiated mESC at later time points during differentiation (beyond day 12), this likely represents differentiation towards other cell types, which also express these genes, at later stages of development [28], [29] (Table S1). The reason for the differences found in PrE differentiation in the absence of Activin-A and Wnt3a in our studies is not readily explained, but may be caused by differences in cytokine content in FCS used in our studies and those of others.

It should be noted that with or without growth factors, the final differentiated progeny from mESC is still mixed. For instance, on day 28 of differentiation, mature hepatic genes (*Tat*, *Pepck*, *G6pc*, *Cyp's*) are expressed combined with a persistent expression of early hepatic genes such as *Afp* and *Ttr*. As we also found high level expression of *Krt19* and *Krt7* at the end of differentiation, this suggests persistent presence of a high percentage of hepatoblast-like cells, consistent with the high number of Hnf4α^+^ cells seen by immunohistology. As seen in miPSC differentiation [30], we have also suggestive evidence that cells with hepatic stellate cell and endothelial features may co-differentiate during the differentiation process. Other differentiation protocols may generate a higher number of rather immature hepatocyte-like cells expressing *Afp* and *Ttr* such as for instance by re-plating cells at definitive endoderm stage [31]. However, the current protocol appears to generate more mature hepatocyte-like cells, expressing e.g. *Tat*, *G6pc* and *Cyp1a2*, as well as liver-specific non-parenchymal cells.

In conclusion, in contrast to hepatic differentiation from hESC that is dependent on the stepwise addition of growth factors, no significant effect on maturation of mESC-progeny was observed when mESC were allowed to differentiate in the absence of cytokine, but in serum-containing medium. These results suggest that differences exist between lineage specific differentiation of mESC and hESC, requiring optimization of different protocols for ESC from either species.

## Materials and Methods

### Media Composition and Cytokines

*Basal differentiation medium:* 60% DMEM-low glucose (Gibco 31885), 40% MCDB-201-water (Sigma M-6770), 0.25× Linoleic acid – Bovine serum albumin (LA-BSA, Sigma L-9530), 0.25× Insulin-transferrin-selenium (ITS, Sigma I-3146), 1× Penicillin-Streptomycin (Cellgro 30-002-CI), 0.1 µM L-Ascorbic Acid (Sigma A8960), 10^−3^ µM Dexamethasone (Sigma D2915), 110 µM 2-mercaptoethanol (Gibco 31350).

*Mouse embryonic fibroblasts (MEFs) expansion medium*: 90% DMEM high glucose (Gibco 41965), 10% fetal bovine serum (FBS, HyClone), 2× L-glutamine (Invitrogen 2503-032), 2× Penicillin-Streptomycin, 2× MEM NEAA (Invitrogen 11140-035), 110 µM 2-mercaptoethanol.

*Mouse ESC expansion medium:* 80% DMEM high glucose, 20% FBS, 1× L-glutamine, 1× Penicillin-Streptomycin, 1× MEM NEAA, 1× sodium pyruvate (Invitrogen 11360), 110 µM 2-mercaptoethanol, 50 µl mLIF (Chemicon ESG-1107).

*Cytokines:* The following cytokines and growth factors (all from R&D Systems) were added during differentiation: rh/m/rActivin-A (338-AC), rhBMP4 (314-BP), rhFGF1 (232-FA), rhFGF2 (233-FB), rhFGF4 (235-F4-025), rmFGF8b (423-F8-025), rmFollistatin-288 (769-FS), rhHGF (294-HGN) and rmWnt3a (1324-WN).

### Cell Line Isolation and Maintenance

*Mouse embryonic fibroblasts*: MEFs were purchased from Global Stem Inc, Rockville, USA. MEFs were maintained in MEF expansion medium and immortalized with 10 ng/ml Mitomycin C (KYOWA Mitomycin 2 mg) at passage 6. Mitomycin-treated MEFs were plated at a density of 35,000 cells/cm^2^ on 0.1% gelatine (Ultrapure water 0.1% gelatine, Chemicon ES-006-B) coated 6 well plates (Corning 3516).

*Murine embryonic stem cells*: The R1 cell line, derived from strain 129, was a kind gift from Prof. P. Carmeliet (K.U.Leuven, Belgium), the Bl6 cell line, derived from strain C57BL/6, was a kind gift from Prof. M. Ko (National Institute of Aging, Baltimore, MD). mESC were plated on mitomycin-treated MEFs in mESC expansion medium, maintained in a 21% O_2_ – 5% CO_2_ – 37°C incubator and split 1∶6–1∶8 every 2–3 days by trypsinization with 0.25% trypsin with EDTA (Gibco 25200056).

*Primary murine hepatocytes*: The isolated mouse hepatocytes were a kind gift from Prof. M. Ott (University of Hannover, Germany).

*Human embryonic stem cells*: The H9 cells (purchased from WiCell, Madison, WI) were cultured as described previously [6]. The differentiation, RT-qPCR and functional tests described previously [6], were currently repeated with removal of cytokines as the only alteration.

#### Hepatic differentiation

All differentiations were done in 12 well plates (Corning 3513) coated with 2% Matrigel (BD 356231) in a 21% O_2_ – 5.8% CO_2_ – 37°C incubator. Prior to starting the hepatic differentiation, undifferentiated mESC were plated for one passage on 0.1% gelatin without MEFs to minimize the contamination with MEFs upon initiation of differentiation. mESC were trypsinized (trypsin 0.25%, Gibco 25200) and re-plated at a density of 12,500–25,000 cells/cm^2^ in basal differentiation medium, combined with the sequential cytokine cocktails as described in Figure 1 for 28 days. In the serum conditions 2% FBS was added for the first 2 days, but this was reduced to 0.5% after 2 days. In the cytokine group, basal differentiation medium was supplemented with 100 ng/ml Activin-A and Wnt3a from d0–6, 10 ng/ml FGF2 and 50 ng/ml BMP4 from d6–10, 25 ng/ml FGF8b, 50 ng/ml FGF1 and 10 ng/ml FGF4 from d10–14 and finally 20 ng/ml HGF and 100 ng/ml Follistatin till d28.

#### RNA isolation and RT-qPCR

Sequences of primers are given in Table S2. As housekeeping gene glyceraldehydes-3-phosphate dehydrogenase (*Gapdh*) was used. Qiagen RNeasy kit was used for RNA isolation as per manufacturer's instruction. Results are expressed as ΔCT values with respect to *Gapdh*, calculated as (CT_gene_ – CT_Gapdh_), and thus the lower the number, the higher the expression level. ΔCT values of >16 are considered as not expressed. In the graphs, relative expression compared to day 0 is depicted, calculated as 2∧(ΔCT_day 0_ – ΔCT_day 6/28_).

#### Immunohistochemistry

Primary and secondary antibodies are given in Table S3. Cells were washed three times with PBS and fixed with 10% neutral buffered formalin (Sigma) for 15 minutes at room temperature (RT). Cells were permeabilized with PBST (0.2% Triton X-100 (Acros Organics) in PBS) for 30 minutes. Next, blocking was performed by incubated the cells with 3% donkey serum (Jackson Labs, JACK017-000-121) in PBST for 30 minutes at RT. After removal of the blocking buffer, cells were incubated overnight at 4°C with the primary antibodies in Dako antibody diluent (Dako S202230). After washing with PBST, cells were incubated with fluorescence-labelled secondary antibody and counterstained with Hoechst diluted in Dako diluent for 2 hours at RT. Quantification of Hnf4α^+^ and Alb^+^ cells was performed using Zeiss AxioVision Software version 4.8.1 on >15 randomly taken pictures.

#### Albumin secretion

On d26, differentiation medium was completely removed and 1.5 ml of fresh medium was added per well. After 48 hours, medium was collected and stored at −80°C until further analysis. Mouse albumin was measured using a quantitative ELISA kit (Starters kit Bethyl E101 and Bethyl E80–129), following the manufacturer's instructions.

#### Urea production

mESC progeny were washed with PBS and cultured with 1 ml of differentiation medium containing 0 or 1 mM NH_4_HCO_3_ for 24 hours. Urea content was calculated using QuantiChrom™ Urea Assay Kit (BioAssay Systems DIUR-500), as per manufacturer's protocol.

#### Glycogen storage

Glycogen content was measured according to the spectrophotometrical method of Seifter et al. [32], as described for rMAPC previously [33]. mESC progeny were scraped from the Matrigel coated wells and collected in 200 µl H_2_O at day 28.

#### Cytochrome P450 activity

Cytochrome 1a2 activity was detected by using the non-lytic method of P450-Glo™ Assay (Promega V8901 and V8771). Induction of Cyp1a2 was performed by incubation 500 µM phenobarbital, as per manufacturer's protocol.

## Supporting Information

Table S1 Gene expression during hepatic differentiation of mESC R1 (Table S1a) and mESC Bl6 (Table S1b) in the presence (+) or absence (−) of growth factors. Results are expressed as ΔCT values ± SD. * significant higher (p<0.05) expressed, n≥3.
(DOC)

Table S2 Mouse (Table S2a) and human primers (Table S2b) for RT-qPCR.
(DOC)

Table S3 Primary and secondary antibodies for immunocytochemistry.
(DOC)
