# Supplementary material for: Culture of Mouse Embryonic Stem Cells with Serum but without Exogenous Growth Factors Is Sufficient to Generate Functional Hepatocyte-Like Cells
Source: PLoS One. 2011 Aug 2;6(8):e23096. doi: 10.1371/journal.pone.0023096 (PMC3149071; doi:10.1371/journal.pone.0023096)
Supplement: Table S1 — Gene expression during hepatic differentiation of mESC R1 (Table S1a) and mESC Bl6 (Table S1b) in the presence (+) or absence (−) of growth factors. Results are expressed as ΔCT values ± SD. * significant higher (p<0.05) expressed, n≥3. (DOC) [file pone.0023096.s001.doc]

**Table S1a**

|  | D0 | D6 | | D12 | | D28 | |  |
| --- | --- | --- | --- | --- | --- | --- | --- | --- |
|  |  | + | - | + | - | + | - |  |
| ***Mesendoderm/definitive endoderm and primitive endoderm*** | | | | | | | | |
| ***Brachyury*** | 13.2 + 3.1 | 3.8 + 1.4 | 4.1 + 1.4 | 9.2 + 1.3 | **4.2 + 1.4*** | - | - |  |
| ***Cxcr4*** | 9.0 + 2.0 | **0.2 + 0.8*** | 4.1 + 0.8 | 2.2 + 1.0 | 2.1 + 2.3 | - | - |  |
| ***Eomes*** | 12.2 + 2 | **4.5 + 1.6*** | 8.7 + 1.7 | 11.5 + 2.0 | 11.1 + 2.2 | - | - |  |
| ***Gsc*** | 11.4 + 1.8 | **1.0 + 1.3*** | 6.9 + 1.0 | 10.2 + 1.3 | 9.0 + 0.1 | - | - |  |
| ***Mixl1*** | 13.0 + 1.3 | **2.8 + 0.9*** | 7.8 + 1.2 | 11.8 + 1.7 | 13 + 1.7 | - | - |  |
| ***Oct4*** | 2.5 + 1.9 | 4.8 + 2.4 | 5.8 + 2.1 | 10.0 + 3.0 | 9.1 + 1.5 | - | - |  |
| ***Sox7*** | 13.5 + 3.1 | **9.2 + 2.5*** | 11.4 + 2.2 | 10.2 + 2.1 | 8.5 + 2.8 | - | - |  |
| ***Sox17*** | NE | **11.3 + 1.4*** | NE | 11.0 + 1.7 | 10.7 + 1.4 | - | - |  |
| ***Thbd*** | 11.4 + 1 | 9.3 + 0.9 | 9.6 + 0.2 | 5.1 + 0.9 | 5.4 + 0.1 | - | - |  |
| ***Tmprss2*** | NE | 10.2 + 0.1 | 11.5 + 1.1 | 10.2 + 1.0 | 11.3 + 2.1 | - | - |  |
| ***Hepatocytes*** | | | | | | | | |
| ***Aat*** | NE | NE | NE | 8.1 + 1.0 | 8.1 + 2.1 | 4.2 + 2.2 | 5.0 + 1.1 |  |
| ***Afp*** | NE | **9.0 + 0.5*** | 15.7 + 3.3 | 3.4 + 1.4 | 6.2 + 4.2 | **-3.1 + 2.8*** | 1.0 + 1.7 |  |
| ***Alb*** | NE | 15.4 + 2.5 | NE | 12.0 + 1.7 | 9.6 + 2.0 | 1.5 + 2.4 | 2.4 + 2.5 |  |
| ***Cyp1a2*** | NE | NE | NE | NE | NE | 8.8 + 2.7 | 9.4 + 2.5 |  |
| ***Cyp7a1*** | NE | NE | NE | NE | NE | 12.6 + 2.3 | 11.0 + 2.5 |  |
| ***Foxa2*** | NE | **9.3 + 0.8*** | 14.1 + 1.8 | 10.6 + 1.0 | 9.8 + 1.6 | **7.0 + 1.4*** | 8.2 + 1.6 |  |
| ***G6pc*** | NE | NE | NE | 16.0 + 2.9 | 15.1 + 1.4 | 9.5 + 1.6 | 9.4 + 1.6 |  |
| ***Hnf4a*** | 11.2 + 3.6 | **6.0 + 1.0*** | 11.1 + 1.8 | **5.1 + 1.3*** | 7.6 + 1.0 | **2.7 + 2.2*** | 5.1 + 0.5 |  |
| ***Krt7*** | 13.0 + 1.4 | 5.4 + 1.0 | 5.7 + 0.5 | 4.8 + 2.3 | 4.5 + 1.3 | 2.3 + 1.5 | 3.1 + 2.7 |  |
| ***Krt19*** | 7.0 + 1.0 | 2.0 + 1.3 | 2.0 + 1.3 | 0.9 + 0.9 | 2.0 + 1.7 | -1.4 + 1.8 | -0.7 + 1.3 |  |
| ***Pepck*** | 16.0 + 3.2 | NE | NE | 13.7 + 1.2 | 14.8 + 3.8 | 8.9 + 2.0 | 9.7 + 2.8 |  |
| ***Tat*** | NE | NE | 15.1 + 1.9 | 10.5 + 0.7 | 11.4 + 1.1 | 7.5 + 2.5 | 5.8 + 2.6 |  |
| ***Ttr*** | NE | 6.0 + 1.0 | 8.7 + 3.1 | **0.6 + 1.4*** | 4.2 + 2.1 | -1.2 + 2.2 | 0.0 + 1.7 |  |
| ***Hepatic stellate cells*** | | | | | | | | |
| ***Alcam*** | 10.7 + 1.2 | 10.2 + 1.2 | 7.9 + 0.0 | 7.4 + 1.6 | **5.2 + 1.2*** | 4.2 + 1.3 | 4.5 + 2.6 |  |
| ***α-sma*** | 6.4 + 0.9 | 6.3 + 1.4 | 3.6 + 0.8 | 4.7 + 2.7 | 2.2 + 1.7 | 3.0 + 2.3 | 1.8 + 2.6 |  |
| ***Col1a1*** | 5.4 + 0.7 | 4.5 + 1.0 | 2.5 + 1.3 | 2.2 + 1.9 | 0.2 + 1.3 | 0.0 + 2.0 | 0.2 + 2.0 |  |
| ***Crbp1*** | 12.7 + 1.0 | 7.8 + 0.6 | 8.2 + 0.9 | 8.5 + 0.5 | 8.8 + 0.8 | 7.4 + 0.5 | 6.3 + 2.2 |  |
| ***Desmin*** | 8.2 + 2.0 | 9.8 + 0.1 | **6.4 + 0.2*** | 6.9 + 1.7 | 4.9 + 1.7 | 5.1 + 2.0 | 4.9 + 1.0 |  |
| ***Gfap*** | NE | NE | NE | NE | 13.8 + 1.8 | 15 + 2 | **6.1 + 3.6*** |  |
| ***Hepatic sinusoidal endothelial cells*** | | | | | | | | |
| ***CD32b*** | 12.9 + 0.5 | NE | NE | NE | NE | 13.8 + 0.1 | 14.8 + 1.3 |  |
| ***Lyve1*** | NE | 15.2 + 0.9 | 13.0 + 0.8 | 13.4 + 0.1 | 11.5 + 3.0 | 9.7 + 0.8 | 9.7 + 1.8 |  |
| ***Mrc1*** | 12.5 + 1.0 | 12.5 + 1.2 | 14.4 + 1.4 | NE | 15.4 + 0.4 | 12.9 + 1.4 | 14.2 + 0.8 |  |
| ***Stab2*** | 15.7 + 1.2 | NE | NE | NE | 15.7 + 1.2 | NE | 14.7 + 1.4 |  |
| ***Tie2*** | 13.8 + 1.2 | - | - | - | - | 9.0 + 1.6 | 9.6 + 1.3 |  |
| ***Vap1*** | 11.4 + 3.7 | 15.1 + 2.4 | 14.4 + 3.4 | 13.5 + 2.5 | 12.4 + 2.8 | 12.1 + 1.7 | 12.0 + 5.2 |  |
| ***Ve-cadherin*** | 12.3 + 1.0 | - | - | - | - | 2.0 + 0.7 | 2.0 + 1.3 |  |
| ***Mesoderm and neuroectoderm*** | | | | | | | | |
| ***Cnn1*** | 10.2 + 2.2 | 9.7 + 1.4 | 5.6 + 3 | 10.6 + 2.8 | 8.6 + 4.8 | 9.9 + 2 | 9.1 + 2.4 |  |
| ***Mesp2*** | 11.5 + 1.2 | 12.4 + 2.5 | 12.2 + 1.8 | 13.6 + 0.7 | 15.4 + 2.7 | 12.4 + 0.4 | 11.6 + 1.8 |  |
| ***Nkx2-5*** | NE | NE | NE | NE | NE | NE | NE |  |
| ***Osterix*** | 12.9 + 0.8 | 14.3 + 1.3 | 15.1 + 1.5 | 13.6 + 0.6 | 11.1 + 1.0 | 10.2 + 2.2 | 8.4 + 1.3 |  |
| ***Sm22*** | 12.8 + 3.2 | 11.3 + 0.6 | 8.9 + 0.6 | 14.8 + 3.1 | 11.5 + 3.3 | 15.6 + 2.5 | 11.2 + 2.7 |  |
| ***Tbx3*** | 7.3 + 0.9 | 6.6 + 1.1 | 7.9 + 1.4 | 8.7 + 0.8 | 9.7 + 1.1 | 6.7 + 0.8 | 7.5 + 0.2 |  |
| ***Otx2*** | 7.0 + 1.4 | **3.3 + 0.9*** | 6.6 + 1.2 | 8.8 + 2.0 | 8.2 + 1.7 | - | - |  |
| ***Pax6*** | 12.3 + 2.1 | 13.8 + 2.6 | 14.6 + 1.6 | 14.2 + 1.1 | 11.7 + 2.6 | - | - |  |
| ***Sox1*** | 12.7 + 0.2 | 14.6 + 0.3 | 12.6 + 1.9 | NE | 11.4 + 2.0 | - | - |  |
| ***Sox2*** | 4.3 + 0.8 | 9.1 + 0.7 | 9.8 + 0.8 | 10.4 + 2.1 | 7.7 + 1.7 | - | - |  |
| ***Growth factors*** | | | | | | | | |
| ***Nodal*** | 4.1 + 1.9 | **4.1 + 1.0*** | 6.6 + 1.4 | 9.5 + 1.3 | 10.7 + 1.3 | 10.4 + 1.8 | 11.4 + 0.9 |  |
| ***Wnt3a*** | NE | NE | NE | NE | 15.0 + 1.4 | 10.5 + 2.4 | 12.8 + 2.1 |  |
| ***Foxh1*** | 4.1 + 2.1 | 4.0 + 1.2 | 5.5 + 2.2 | 9.2 + 2.3 | 9.1 + 1.7 | 11.4 + 3.5 | 10.7 + 1.6 |  |
| ***Bmp4*** | 3.8 + 1.4 | 4.4 + 1.3 | 4.0 + 2.1 | 6.3 + 0.8 | 6.1 + 1.7 | 7.5 + 2.3 | 6.5 + 1.2 |  |
| ***Fgf2*** | 7.2 + 2.3 | 11.4 + 1.4 | 10.6 + 0.7 | 10.2 + 2.8 | 10.6 + 0.9 | 8.6 + 0.4 | 8.6 + 0.8 |  |
| ***Fgf4*** | 3.8 + 0.4 | 10.3 + 1.3 | 9.3 + 0.6 | 13.3 + 1.3 | 10.9 + 1.4 | 13.2 + 1.4 | 11.2 + 2.3 |  |
| ***Fgf8*** | 11.6 + 2.7 | **3.6 + 1.2*** | 7.3 + 1.4 | 12.1 + 2.4 | 11.7 + 0.5 | 13.3 + 1.1 | 12.3 + 1.7 |  |
| ***Hgf*** | 13.0 + 2.2 | 14.0 + 0.9 | 14.5 + 2.0 | 10.0 + 1.1 | 13.0 + 1.1 | 10.3 + 0.7 | 12.4 + 2.2 |  |

**Table S1b**

|  | D0 | D6 | | D12 | | D28 | |  |
| --- | --- | --- | --- | --- | --- | --- | --- | --- |
|  |  | + | - | + | - | + | - |  |
| ***Mesendoderm/definitive endoderm and primitive endoderm*** | | | | | | | | |
| ***Brachyury*** | 12.7 + 3.2 | **3.7 + 1.2*** | 5.9 + 1.3 | 9.0 + 3.1 | 7.2 + 1.5 | - | - |  |
| ***Cxcr4*** | 8.0 + 2.1 | **2.2 + 0.5*** | 5.2 + 1.4 | 3.1 + 1.7 | 4.0 + 1.0 | - | - |  |
| ***Eomes*** | 11.9 + 1.6 | **7 + 1.3*** | 10.8 + 2.7 | 12.8 + 2 | 11.5 + 2.3 | - | - |  |
| ***Gsc*** | 12.2 + 1.7 | **3.5 + 1.1*** | 8.8 + 2.1 | 11.6 + 1.5 | 9.8 + 2 | - | - |  |
| ***Mixl1*** | 12.4 + 1.5 | **4.8 + 0.6*** | 9.9 + 2 | 14.1 + 1.6 | 13.7 + 1.3 | - | - |  |
| ***Oct4*** | 3.3 + 2.0 | **4.0 + 2.4*** | 7.0 + 1.6 | 9.0 + 2.2 | 8.5 + 1.9 | - | - |  |
| ***Sox7*** | 11.8 + 3.0 | 11.9 + 1.5 | 11.9 + 4.0 | 10.7 + 4.2 | 5.9 + 4.5 | - | - |  |
| ***Sox17*** | NE | 13.8 + 3.3 | 15.2 + 3.2 | 11.3 + 3.1 | 15.5 + 1.4 | - | - |  |
| ***Thbd*** | 11.4 + 0.2 | 8.5 + 1.1 | 8.4 + 0.3 | 4.7 + 0.2 | 5.1 + 1.3 | - | - |  |
| ***Tmprss2*** | NE | 11.2 + 0.1 | 11.1 + 0.4 | 9.9 + 1.5 | 11.1 + 1.4 | - | - |  |
| ***Hepatocytes*** | | | | | | | | |
| ***Aat*** | 14.0 + 5.0 | NE | NE | 6.4 + 2.9 | 7.6 + 2.0 | 2.8 + 2.4 | 4.1 + 3.2 |  |
| ***Afp*** | NE | **12.0 + 3.3*** | 16 + 2.1 | **5.2 + 2.0*** | 8.0 + 2.5 | -**1.2 + 3.0*** | 0.5 + 3.0 |  |
| ***Alb*** | NE | NE | NE | 10.0 + 1.7 | 8.4 + 1.2 | 2.0 + 3.0 | 2.6 + 3.1 |  |
| ***Cyp1a2*** | NE | NE | NE | NE | NE | 7.3 + 0.8 | 8.7 + 3.1 |  |
| ***Cyp7a1*** | NE | NE | NE | 13.7 + 1.7 | 13.0 + 2.6 | 10.7 + 1.7 | 10.8 + 3.2 |  |
| ***Foxa2*** | NE | **10.1 + 1.2*** | 13.1 + 1.8 | **10.1 + 2.0*** | 12.5 + 2.0 | 7.9 + 2.1 | 7.7 + 1.9 |  |
| ***G6pc*** | NE | NE | NE | NE | 13.6 + 1.6 | 9.0 + 1.4 | 8.3 + 2.4 |  |
| ***Hnf4a*** | 15.1 + 1.3 | **6.7 + 2.1*** | 11.7 + 3.1 | **6.7 + 1.2*** | 10.7 + 0.8 | 3.8 + 2.0 | 5.1 + 0.8 |  |
| ***Krt7*** | 12.1 + 1.2 | 6.2 + 2.7 | 4.6 + 1.7 | 4.5 + 1.8 | 3.7 + 1.3 | 2.3 + 1.0 | 4.9 + 2.2 |  |
| ***Krt19*** | 7.4 + 2.4 | -0.26 + 2.0 | 0.5 + 1.8 | 1.0 + 1.2 | 1.3 + 2.3 | -1.5 + 2.4 | -0.1 + 1.3 |  |
| ***Pepck*** | NE | NE | NE | 14.3 + 1.8 | 13.7 + 3.1 | 8.5 + 2.0 | 9.2 + 3.5 |  |
| ***Tat*** | 15.8 + 1.7 | NE | NE | 10.9 + 1.8 | 10.9 + 0.7 | 5.1 + 4.2 | 5.5 + 3.1 |  |
| ***Ttr*** | NE | **7.8 + 2.9*** | 9.6 + 3.0 | **2.2 + 2.6*** | 6.9 + 1.0 | 0.6 + 2.2 | -1.1 + 2.3 |  |
| ***Hepatic stellate cells*** | | | | | | | | |
| ***Alcam*** | 10.1 + 1.1 | 9.5 + 1.5 | 8.1 + 0.7 | 8.8 + 2.4 | 6.9 + 0.6 | 4.4 + 1.9 | 5.8 + 1.9 |  |
| ***α-sma*** | 6.1 + 1.0 | 5.18 + 1.0 | 3.8 + 1.2 | 5.5 + 1.8 | **2.7 + 1.3*** | 3.3 + 1.4 | 3.3 + 3.0 |  |
| ***Col1a1*** | 5.4 + 1.1 | 3.8 + 1.6 | 2.2 + 0.8 | 2.5 + 1.5 | 1.3 + 1.0 | 1.1 + 1.3 | 0.9 + 1.7 |  |
| ***Crbp1*** | 12.1 + 2.2 | 7.9 + 1.0 | 6.9 + 0.1 | 7.1 + 0.7 | 7.4 + 0.5 | 6.8 + 0.5 | 7.3 + 1.3 |  |
| ***Desmin*** | 8.3 + 0.3 | 9.1 + 2.3 | 6.1 + 1.3 | 7.7 + 1.0 | 6.0 + 1.6 | 7.1 + 1.1 | 7.0 + 1.7 |  |
| ***Gfap*** | NE | NE | NE | NE | 15.2 + 0.0 | 12.0 + 4.2 | 7.8 + 5.8 |  |
| ***Hepatic sinusoidal endothelial cells*** | | | | | | | | |
| ***CD32b*** | 13.5 + 1.4 | NE | NE | NE | NE | 12.3 + 2.8 | 11.6 + 2.8 |  |
| ***Lyve1*** | NE | NE | NE | 11.1 + 0.4 | 12.1 + 0.7 | 9.7 + 0.5 | 10.0 + 0.7 |  |
| ***Mrc1*** | 14.9 + 0.2 | 14.5 + 1.4 | 14.0 + 1.4 | NE | NE | 11.8 + 0.6 | 11.2 + 1.9 |  |
| ***Stab2*** | NE | NE | NE | 13.0 + 0.0 | 15.8 + 1.2 | 15.4 + 0.8 | 12.4 + 1.6 |  |
| ***Vap1*** | 13.9 + 3 | 15.3 + 3.9 | 13.2 + 3.2 | 9.8 + 1.1 | 9.6 + 2.3 | 12.2 + 2.0 | 9.8 + 1.8 |  |
| ***Mesoderm and neuroectoderm*** | | | | | | | | |
| ***Cnn1*** | 10.5 + 1.0 | 8.2 + 2.1 | 6.1 + 2.3 | 10.3 + 1.0 | **8.2 + 0.6*** | 8.6 + 1.7 | 7.7 + 0.4 |  |
| ***Mesp2*** | 13.3 + 0.7 | 13.9 + 2.1 | 14.2 + 1.1 | NE | NE | 15.3 + 1.1 | 13.7 + 1.1 |  |
| ***Nkx2-5*** | NE | NE | NE | NE | NE | NE | NE |  |
| ***Osterix*** | 14.8 + 0.7 | NE | NE | 14.9 + 4.5 | 14.0 + 0.5 | 13.6 + 2.8 | 13.5 + 2.8 |  |
| ***Sm22*** | 11.5 + 0.5 | 11.1 + 2.2 | 8.5 + 0.2 | 14.4 + 2.6 | 14.9 + 3.1 | 12.3 + 3.1 | 11.7 + 2 |  |
| ***Tbx3*** | 7.3 + 0.9 | 6.6 + 1.1 | 7.9 + 1.4 | 8.7 + 0.8 | 9.7 + 1.1 | 6.7 + 0.8 | 7.5 + 0.2 |  |
| ***Otx2*** | 9.2 + 3.5 | 5.0 + 1.7 | 8.8 + 2.5 | 10.2 + 1.9 | 9.7 + 1.5 | - | - |  |
| ***Pax6*** | 13.8 + 0.7 | 15.7 + 0.6 | 15.3 + 0.8 | 14.1 + 1.1 | 11.6 + 1.4 | - | - |  |
| ***Sox1*** | 11.5 + 0.4 | 15.0 + 1.7 | 13.6 + 0.7 | 15.6 + 4.0 | 10.7 + 0.4 | - | - |  |
| ***Sox2*** | 5.1 + 1.3 | 9.9 + 0.6 | 8.9 + 1.2 | 10.1 + 0.7 | 9.0 + 1.7 | - | - |  |
| ***Growth factors*** | | | | | | | | |
| ***Nodal*** | 3.6 + 1.5 | 3.1 + 2.5 | 4.7 + 1.3 | 8.3 + 1.0 | 9.3 + 1.2 | 9.3 + 1.3 | 12.2 + 4.1 |  |
| ***Wnt3a*** | 15.6 + 1.7 | NE | NE | NE | 16.0 + 1.1 | 13.8 + 1.8 | 14.9 + 1.6 |  |
| ***Foxh1*** | 4.8 + 1.7 | 2.8 + 2.1 | 3.9 + 1.8 | 8.6 + 2.0 | 8.2 + 0.9 | 11.1 + 2.1 | 9.9 + 2.2 |  |
| ***Bmp4*** | 7.0 + 1.6 | 6.5 + 2.4 | 5.9 + 1.5 | 8.6 + 0.6 | 8.2 + 1.1 | 8.2 + 1.5 | 6.5 + 1.5 |  |
| ***Fgf4*** | 3.9 + 1.0 | 6.7 + 1.0 | 7.5 + 1.0 | 12.1 + 1.7 | 10.3 + 1.7 | 13.4 + 3.8 | 12.4 + 4.0 |  |
| ***Fgf8*** | 11.3 + 3.3 | **3.0 + 2.2*** | 5.4 + 2.0 | 13.3 + 2.7 | 9.8 + 2.0 | 12.8 + 2.8 | 11.7 + 1.1 |  |
| ***Hgf*** | 14.4 + 2.8 | 14.8 + 2.7 | 12.5 + 1.4 | 11.5 + 1 | 10.7 + 2.0 | 10.7 + 2.5 | 11.1 + 2.6 |  |
