# Supplementary material for: Culture of Mouse Embryonic Stem Cells with Serum but without Exogenous Growth Factors Is Sufficient to Generate Functional Hepatocyte-Like Cells
Source: PLoS One. 2011 Aug 2;6(8):e23096. doi: 10.1371/journal.pone.0023096 (PMC3149071; doi:10.1371/journal.pone.0023096)
Supplement: Table S2 — Mouse (Table S2a) and human primers (Table S2b) for RT-qPCR. (DOC) [file pone.0023096.s002.doc]

**Table S2a**

| **Genes** | **Forward sequence** | **Reverse sequence** |
| --- | --- | --- |
| **αsma** | CGCTGTCAGGAACCCTGAGA | CGAAGCCGGCCTTACAGA |
| **Aat** | TTAAAGGCAAATGGAAGAAGCC | GCATAATCCATCAGCAGCAC |
| **Afp** | GAGTGCTTCCAGACAAAGAG | ATAATGGTTGTTGCCTGGAG |
| **Alb** | AGACTGCCTTGTGTGGAAGACT | TCAACTGTCAGAGCAGAGAAGC |
| **Alcam** | CTCGTTGCTGGTGTCGTCTA | AATCCGCTCCTCTCTTAGGC |
| **Bmp4** | TTTCCATCACGAAGAACATCTG | GGAGATCACCTCATTCTCTG |
| **Brachyury** | TCCCGAGACCCAGTTCATAG | TTCTTTGGCATCAAGGAAGG |
| **CD32b** | TGTTCTCACGGACTTTGTGC | CTCGAGTTTGACCACAGCCT |
| **Cnn1** | ACATCATTGGACTGCAGATG | CAAAGATCTGCCGCTTGGTG |
| **Col1a1** | CACCACCCTCAAGAGCCTGAGTC | GTTCGGGCTGATGTACCAGT |
| **Crbp1** | CCAGACAAAGAGATCGTGCAG | CACACACTGGAGTTTGTCACC |
| **Cxcr4** | GCCATGGCTGACTGGTACTT | TTTCAGCCAGCAGTTTCCTT |
| **Cyp1a2** | GGAGCACGTGAGCAAGGAGG | ACCTCTTGAGGGCCGGGTT |
| **Cyp7a1** | GAGCCCTGAAGCAATGAAAG | GCTGTCCGGATATTCAAGGA |
| **Desmin** | CAGGACCTGCTCAATGTGAA | GTAGCCTCGCTGACAACCTC |
| **Eomes** | GGCCTACCAAAACACGGATATC | TTTCTGAAGCCGTGTACATGGA |
| **Fgf2** | GTCAAACTACAACTCCAAGCA | GTCGTTCAAAGAAGAAACACTC |
| **Fgf4** | CCAACAACTACAACGCCTACGA | CCTTCTTGGTCCGCCCGTTC |
| **Fgf8** | AGACAGGTCTCTACATCTGC | GTGTAGTTGTTCTCCAGCAC |
| **Foxa2** | CGAGTTAAAGTATGCTGGGAG | TATGTGTTCATGCCATTCATCC |
| **Foxh1** | TGGCTCAGATTATCCGTCAG | CCGATTAGAGGAAAGGTTGTG |
| **Gapdh** | AAGGGCTCATGACCACAGTC | GGATGCAGGGATGATGTTCT |
| **Gfap** | CACGAACGAGTCCCTAGAGC | CCTTCTGACACGGATTTGGT |
| **Gsc** | GAGAACCTCTTCCAGGAGAC | TTCTTAAACCAGACCTCCACC |
| **G6pc** | TCTTCAAGTGGATTCTGTTTGG | GACAGGGAACTGCTTTATTATAGG |
| **Hgf** | TTCCAGCCAGAAACAAAGAC | GAGCCTTCAGGACCATAGAC |
| **Hnf4α** | GGTCAAGCTACGAGGACAGC | ATGTACTTGGCCCACTCGAC |
| **Krt7** | GGGATGACCTCCGCAACACC | CTCCAGCAGCTTGCGGTAGG |
| **Krt19** | ACCCTCCCGAGATTACAACC | CAAGGCGTGTTCTGTCTCAA |
| **Lyve1** | AGGAGCCCTCTCCTTACTGC | ACCTGGAAGCCTGTCTCTGA |
| **Mesp2** | ATCCACTGAACCTGCAGAGC | GGCTGTAGTCTCTGGCATGA |
| **Mixl1** | CTACCCGAGTCCAGGATCCA | ACTCCCCGCCTTGAGGATAA |
| **Mrc1** | TGCATTGGTTTGTCCTTTTTC | GCAGGGTTGACATGAGACCT |
| **Nkx2-5** | GCGTCGGGGACTTGAACACC | GCGCACTCACTTTAATGGGAAG |
| **Nodal** | GTACATGTTGAGCCTCTACC | GAGAAGTCAAACGTGAAAGTC |
| **Oct4** | CCAATCAGCTTGGGCTAGAG | CCTGGGAAAGGTGTCCTGTA |
| **Osterix** | AGCGACCACTTGAGCAAACAT | GCGGCTGATTGGCTTCTTCT |
| **Otx2** | AGGACGACATTTACTAGGGC | GATTCTTAAACCATACCTGCACC |
| **Pax6** | TCAGACCTCCTCATACTCGTGCA | TGTAGGTATCATAACTCCGCCCA |
| **Pepck1** | CATTGAGGGTATCATCTTTGG | ATGATGATCTTGCCCTTGTG |
| **Sm22** | CCACAAACGACCAAGCCTTCT | CGGCTCATGCCGTAGGAT |
| **Sox1** | AACTCTCAGGGCTACATGAG | GACTTGACCAGAGATCCGAG |
| **Sox2** | AGGAAGGAGTTTATTCGGATTTG | ACGATATCAACCTGCATGGAC |
| **Sox7** | TGTTGACAGATCCCCAAGAA | CTGGGCACCAGTCAATTACA |
| **Sox17** | CATGGATACAATGAGCAGCAC | GCCTTTCACCTTTACATCCC |
| **Stab2** | GACCGGAGTTCTCATTCCTCC | GTGAAGATTGCCGTGGGCT |
| **Tat** | TTAAGTCCAATGCGGACCTC | GCTCTGTGAATTCCACGTCA |
| **Tbx3** | CGAAATGCCAAAGAGGATGT | CTGTTTCCGGGAACAGGTAG |
| **Thbd** | CTTGTGCAATAGGAGCACGA | AAATGCTCGCAGAGTTCGTT |
| **Tmprss2** | AGGTTTACTCATCTCAGAGG | AAAGCTGTTCTTGTATCCCA |
| **Ttr** | CTTTGCCTCTGGGAAGACC | CAGAGTCGTTGGCTGTGAAA |
| **Vap1** | ACCGCATCCAGATACTCAGC | ATGAGGGATGTGCAAAAAGC |
| **Wnt3a** | CACCACCGTCAGCAACAG | TCACTGCGAAAGCTACTCCA |

**Table S2b**

| **Genes** | **Forward sequence** | **Reverse sequence** |
| --- | --- | --- |
| **AFP** | CCTACAATTCTTCTTTGGGCT | AGTAACAGTTATGGCTTGGA |
| **ALB** | TGGCACAATGAAGTGGGTAA | CTGAGCAAAGGCAATCAACA |
| **CXCR4** | AACTTCAGTTTGTTGGCTGC | GAAACAGGGTTCCTTCATGG |
| **FOXA2** | ATTGCTGGTCGTTTGTTGTG | TACGTGTTCATGCCGTTCAT |
| **GSC** | TCTCAACCAGCTGCACTGTC | CCAGACCTCCACTTTCTCCTC |
| **G6PC** | GTGTCCGTGATCGCAGACC | GACGAGGTTGAGCCAGTCTC |
| **HNF4α** | TGTACTCCTGCAGATTTAGCC | CTGTCCTCATAGCTTGACCT |
| **MIXL1** | GGATCCAGGTATGGTTCCAG | CATGAGTCCAGCTTTGAACC |
| **SOX17** | CGCACGGAATTTGAACAGTA | GGATCAGGGACCTGTCACAC |
