# Supplementary material for: Culture of Mouse Embryonic Stem Cells with Serum but without Exogenous Growth Factors Is Sufficient to Generate Functional Hepatocyte-Like Cells
Source: PLoS One. 2011 Aug 2;6(8):e23096. doi: 10.1371/journal.pone.0023096 (PMC3149071; doi:10.1371/journal.pone.0023096)
Supplement: Table S3 — Primary and secondary antibodies for immunocytochemistry. (DOC) [file pone.0023096.s003.doc]

**Table S3**

Primary antibodies

| **Name** | **Company** | **Catalog no.** | **Dilution** | **Isotype** |
| --- | --- | --- | --- | --- |
| ALB | Dako | A0001 | 1:8000 | Rabbit IgG |
| HNF4α | Abcam | AB41898 | 1:200 | Mouse IgG_2A_ |
| MIXL1 | Santa Cruz. Biotech | SC98665 | 1:100 | Rabbit IgG |
| OCT4 | Santa Cruz. Biotech | SC8628 | 1:1000 | Goat IgG |

**Isotypes (protein concentration identical to primary antibody)**

| Mouse IgG_2A_ | Sigma | M9144 |
| --- | --- | --- |
| Goat IgG | Jackson Labs | JACK005-000-0020 |
| Rabbit serum | Dako | X0902 |

Secondary antibodies

| **Name** | **Company** | **Catalog no.** | **Dilution** |
| --- | --- | --- | --- |
| Donkey anti-rabbit Alexa 488 (green) | Invitrogen | A-21206 | 1:500 |
| Donkey anti-goat Alexa 555 (red) | Invitrogen | A-21432 | 1:500 |
| Donkey anti-mouse Alexa 555 (red) | Invitrogen | A-31570 | 1:500 |
| Hoechst (nuclear staining, blue) | Sigma | 33258 | 1:2000 |
